# The ALK inhibitor AZD3463 effectively inhibits growth of sorafenib-resistant acute myeloid leukemia

**DOI:** 10.1038/s41408-018-0169-1

**Published:** 2019-01-15

**Authors:** Sausan A. Moharram, Kinjal Shah, Fatima Khanum, Lars Rönnstrand, Julhash U. Kazi

**Affiliations:** 1 Division of Translational Cancer Research, Department of Laboratory Medicine, Lund University, Lund, Sweden; 2 Lund Stem Cell Center, Department of Laboratory Medicine, Lund University, Lund, Sweden; 3 Division of Oncology, Skåne University Hospital, Lund, Sweden

**Keywords:** Acute myeloid leukaemia, Oncogenes

Acute leukemia is a group of aggressive hematological disorders originating from the myeloid and lymphoid lineages. Although the current combination of chemotherapies has significantly improved the survival of patients with acute lymphoblastic leukemia (ALL), patients with acute myeloid leukemia (AML) still suffer from poor survival^1,2^. Around 30% of AML patients carry an internal tandem duplication (ITD) mutation in the type III receptor tyrosine kinase FLT3 and this group of patients have poor prognosis compared to other patient groups^3,4^. In addition, a small group of ALL patients also carry activating mutations in FLT3^5^. In the past decades, several inhibitors targeting FLT3 have been tested in clinical trials. However, a majority of FLT3 inhibitors displayed transient response as mono-therapy, probably due to the fact that only FLT3 inhibition is not enough for complete remission and due to acquired resistance to the specific inhibitor^6^. In order to find a novel therapy for FLT3-ITD-dependent AML, we used cell lines derived from the AML cell line MOLM-13 that are either sensitive or resistant to the multikinase inhibitor sorafenib. The resistant cell line was generated by long-term treatment of MOLM-13 cells with sorafenib^7^. Sorafenib-resistant cells acquired a secondary mutation in the kinase domain of FLT3 (D835Y) and also displayed upregulation of the PI3K/mTOR pathway. We first characterized sorafenib-sensitive and resistant cells with respect to tyrosine kinase signaling using peptide-based kinase profiling. We observed that peptide substrates selective for PDGFRB, CSK, and FES displayed elevated tyrosine phosphorylation in sorafenib-resistant cells compared to sorafenib-sensitive cells (Fig. S1A, B). Furthermore, treatment of cells with sorafenib inhibited tyrosine phosphorylation of those peptide substrates in sorafenib-sensitive cells but not in sorafenib-resistant cells (Fig. S1C, D). These findings suggest that tyrosine kinases phosphorylate several substrates selective for PDGFRB, CSK, and FES that are involved sorafenib resistance. However, experiments using exome sequencing, RNAseq, and microarray analysis did not show any mutations or overexpression of PDGFRB, CSK, and FES (data not shown).

To determine the kinase-dependency of sorafenib-sensitive and -resistant cells we used a library of 378 kinase inhibitors. Two concentrations, 100 and 1000 nM, were used to treat cells. A cell line of lymphoid lineage was used as a control. We observed that several inhibitors targeting protein tyrosine kinases specifically inhibited the growth of both sorafenib-sensitive and -resistant cells at 100 nM concentration (Fig. S2A, B) as well as at 1000 nM (Fig. S2C, D). Among several potent tyrosine kinase inhibitors, the ALK inhibitor AZD3463 inhibited both sorafenib-sensitive and -resistant cells equally well and was therefore chosen for further analysis (Fig. 1a and S2E). AZD3463 has shown promising result in preclinical testing of neuroblastomas that carry activating ALK mutations^8^. First, we determined the EC_50_ of AZD3463 for both sorafenib-sensitive and -resistant cells. The inhibitor showed an EC_50_ value of around 30 nM for both sorafenib-sensitive and resistant MOLM-13 cells (Fig. S3A) while the resistant cell lines displayed substantial resistance to other FLT3 inhibitors such as AC220, Crenolanib, and PKC412 (data not shown). Furthermore, AZD3463 inhibited the growth of MOLM-13 cells (Fig. S3B) and induced apoptosis (Fig. S3C) in a dose-dependent manner.

**Fig. 1:**
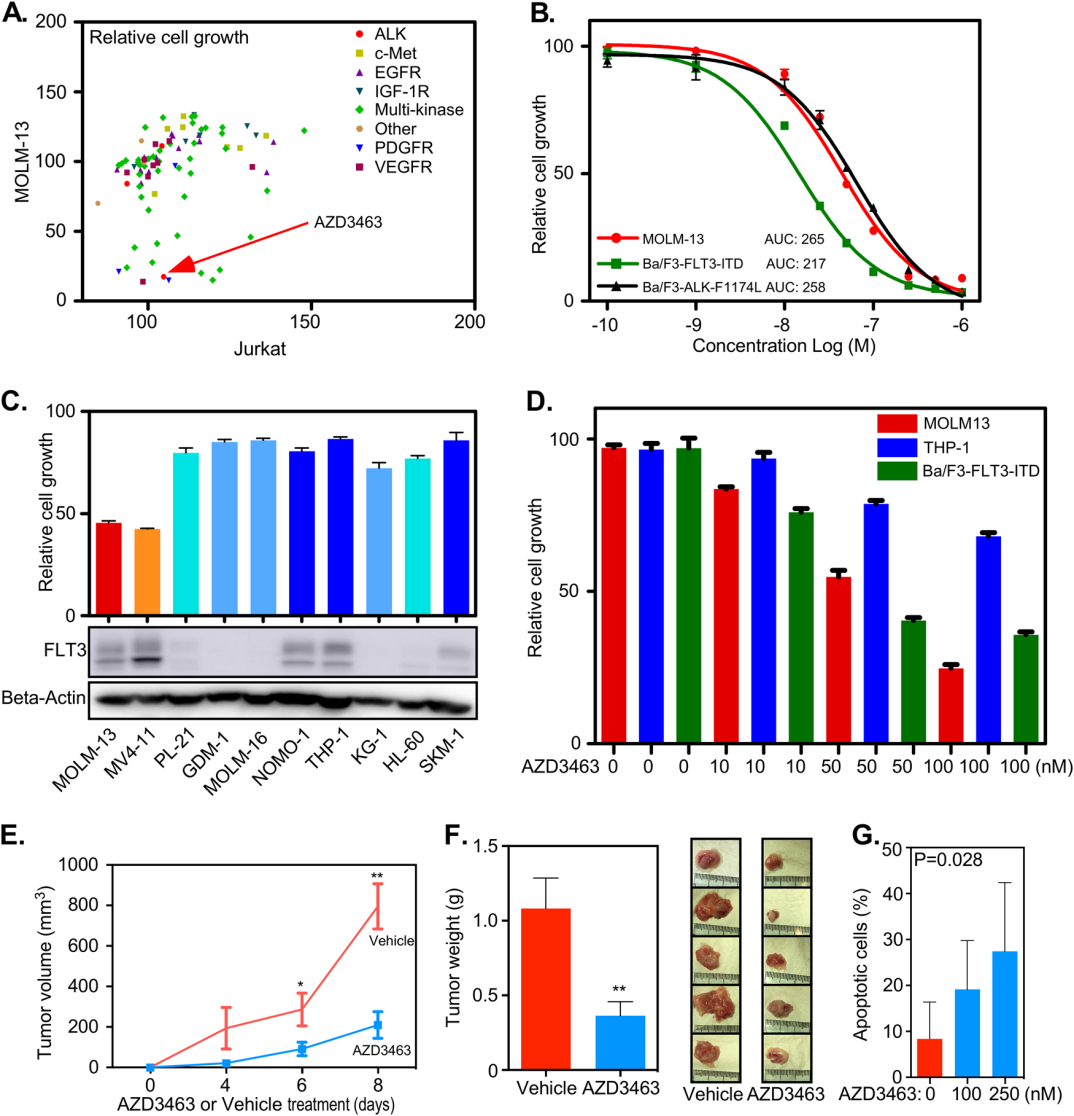
AZD3463 inhibits in vitro cell growth and in vivo tumor formation. **a** MOLM-13 and Jurkat cells were treated with 100 nM of different inhibitors against kinases. Relative cell viability was measured by PrestoBlue after 48 h incubation with the drug. **b** MOLM-13, Ba/F3-FLT3-ITD, and Ba/F3-ALK-F1174L were treated with different concentration of AZD3463 for 48 h. Cell viability was measured by PrestoBlue. Area under the curve (AUC) was determined by GraphPad. **c** Relative cell growth of different AML cell lines was measured in the presence of AZD3463 after 48 h incubation with PrestoBlue. For FLT3 expression, cells were lysed followed by SDS-PAGE analysis. Anti-FLT3 antibody was used for Western blotting. **d** MOLM-13, THP-1, and Ba/F3-FLT3-ITD cells were treated with increasing concentration of AZD3463 for 48 h before measuring cell viability using PrestoBlue. **e** Four million cells were injected subcutaneously into ten mice. One week after injection, half of them were treated with the vehicle while half of them were treated with 15 mg/kg AZD3463. Tumor volume was measured on day 4, 6, and 8 after treatment. **f** Tumor weight was measured after sacrificing mice on the 8th day after treatment. **g** AML patient samples (three) were treated with AZD3463 for 48 h. Apoptosis was measured by Annexin V and 7-AAD kit

Since AZD3463 is an ALK inhibitor; we first analyzed ALK expression in AML cell lines. ALK expression was detected in several AML cell lines, such as MOLM-13, MV4-11, and THP-1 cell lines, but not in the murine proB cell line Ba/F3 or in the murine myeloid cell line 32D (Fig. S4A). However, we were unable to detect any activation of ALK in those AML cell lines (data not shown). Thus, it is likely that cell death induced by AZD3463 was not due to the inhibition of ALK. Tyrosine kinases display a high degree of structural homology in the kinase domain and therefore inhibitors targeting the ATP-binding site are many times not highly specific for a single kinase. Since MOLM-13 cells are dependent on oncogenic FLT3-ITD signaling, and since AZD3463 induced apoptosis as well as growth inhibition, we hypothesized that AZD3463 might inhibit FLT3. To check whether AZD3463 fits to the ATP-binding pocket in FLT3, we used molecular docking. Data demonstrate that AZD3463 is able to occupy the ATP-binding pocket of FLT3 (Fig. S4B) which also overlaps considerably with the binding site of AC220 (Fig. S4C), the most potent and selective inhibitor of FLT3 to date^9^. To further verify the specificity of AZD3463 for FLT3, we generated a Ba/F3 cell line expressing a constitutively active mutant of FLT3 (FLT3-ITD). Ba/F3 cells expressing a constitutively active mutant of ALK (ALK-F1174L) were used as positive control. We observed that Ba/F3 cells expressing FLT3-ITD were more sensitive to AZD3463 compared to cells expressing ALK-F1174L (Fig. 1b). Taken together, these data suggest that AZD3463 is a potent inhibitor of FLT3-ITD as well as of FLT3-ITD carrying a secondary D835Y mutation.

To verify the specificity of AZD3463 toward FLT3-ITD, we used a panel of AML cells expressing wild-type FLT3 or FLT3-ITD. We observed that AZD3463 selectively inhibited MOLM-13 and MV4-11 cells (Fig. 1c), which both express and are dependent on FLT3-ITD, demonstrating that AZD3463 is a selective inhibitor of FLT3-ITD. AZD3463 inhibited the growth of MOLM-13 and Ba/F3-FLT3-ITD cells in a dose-dependent manner but not THP-1 cells (expressing wild-type FLT3) (Fig. 1d). In addition to the in vitro inhibition of FLT3-ITD, we observed that AZD3463 efficiently delayed tumor growth both in terms of tumor volume (Fig. 1e) and weight (Fig. 1f) in a MOLM-13 mouse xenograft model. The inhibitor also induced apoptosis in FLT3-ITD positive primary AML cells in a dose-dependent manner (Fig. 1g). Thus, we suggest that AZD3463 is an active drug against FLT3-ITD-dependent AML.

Wild-type FLT3 is required for normal hematopoiesis^4^. Therefore, inhibition of wild-type FLT3 is likely to cause unwanted side effects. For this reason, we checked whether AZD3463 also inhibits wild-type FLT3 signaling. Ligand-induced activation of FLT3 results in activation of AKT and ERK1/2 signaling. In contrast, FLT3-ITD is constitutively active and activates downstream signaling cascades^10^. We observed that treatment with AZD3463 inhibited FLT3-ITD-mediated activation of AKT, ERK1/2, and p38 in a dose-dependent manner in MOLM-13 cells (Fig. 2a) as well as in MV4-11 cells (Fig. 5SA). Tyrosine phosphorylation of FLT3 was also reduced in a similar fashion (Fig. 2A and Fig. S5A). Both MOLM-13 and MV4-11 carry an FLT3-ITD mutation, but MOLM-13 cells also express one copy of wild-type FLT3. However, AZD3463 did not affect FL-stimulated tyrosine phosphorylation of FLT3 or its downstream signaling in MOLM-13 cells (Fig. 2a and Fig. S5B), in Ba/F3 cells transfected with FLT3-WT (Fig. 2b) or in THP-1 cells (expressing wild-type FLT3; Fig. S5C) suggesting that AZD3463 selectively inhibits oncogenic FLT3-ITD but not wild-type FLT3.

**Fig. 2:**
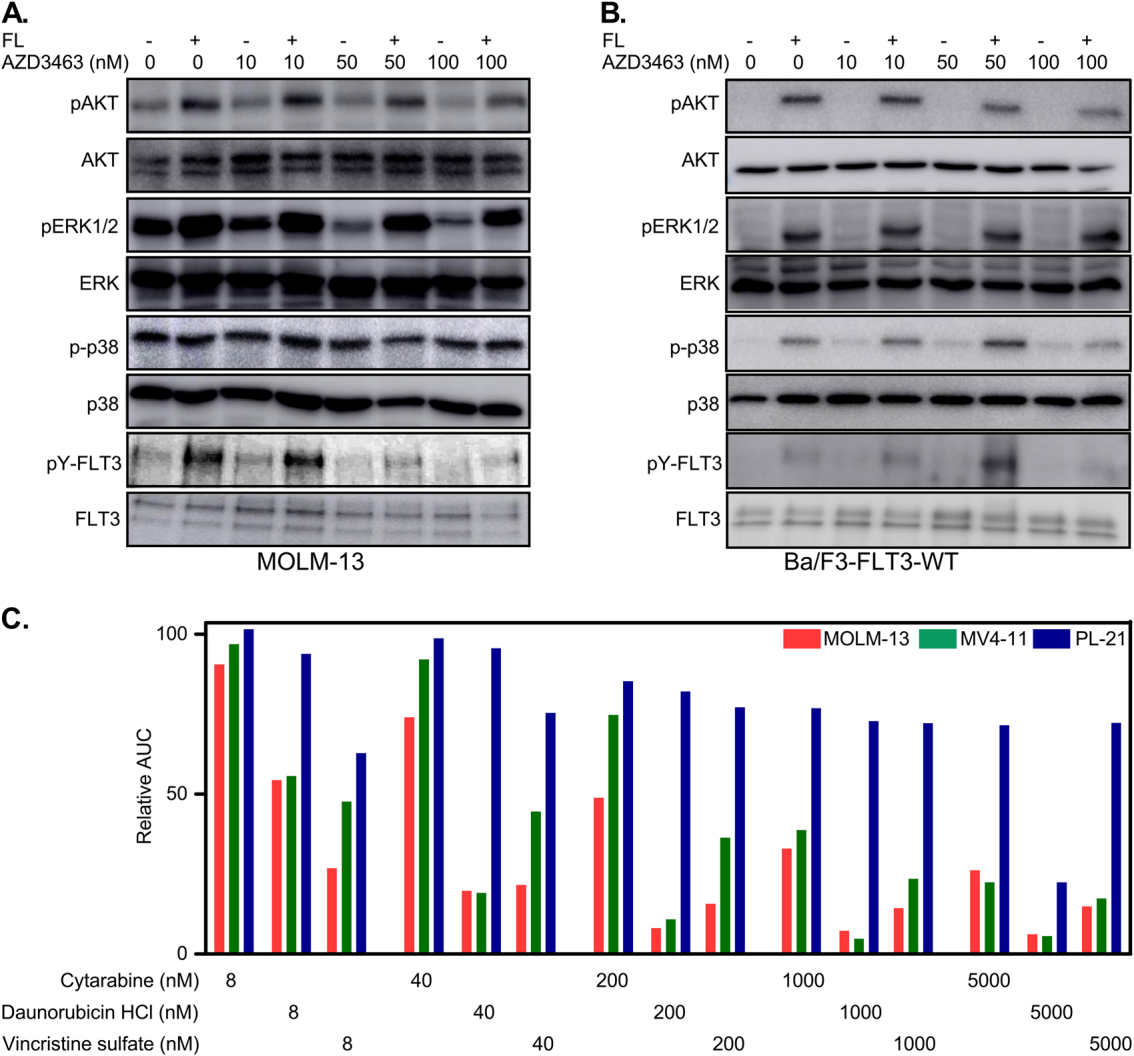
AZD3463 selectively inhibits FLT3-ITD but not ligand-induced wild-type FLT3: **a**, **b** MOLM-13 (**a**) and Ba/F3-FLT3-WT (**b**) cells were treated with different concentrations of AZD3463 for 4 h before stimulation with 100 ng/ml FL for 5 min. Cells were then lysed and lysates were used for anti-FLT3 immunoprecipitation and Western Blot analysis using specific antibodies. **c** Cells were treated with different concentrations of cytarabine or daunorubicin or vincristine and AZD3463. After 2 days cell viability was measured by PrestoBlue cell viability assay. AUC was measured by GraphPad

The use of FLT3 inhibitor as mono-therapy has shown disappointing results in clinical trials^6^. However, in combination with conventional chemotherapy the multispecific kinase inhibitor midostaurin (PKC412), that also inhibits FLT3, displayed promising results and recently received approval by the FDA^11^. To verify whether it would be possible to combine AZD3463 with chemotherapeutic agents, we have tested the effect of AZD3463 in combination with eight chemotherapeutic agents in MOLM-13 and MV4-11 cell lines. PL-21 cells, that did not show any response to AZD3463, was used as a control. We observed that addition of either cytarabine, daunorubicin (or doxorubicin), or vincristine induced an additive effect (decreased area under the curve with increasing concentrations) while the addition of cyclophosphamide, methotrexate, 6-mercaptopurine, or dexamethasone did not show any difference (Fig. 2c, S6, and S7). Cytarabine and daunorubicin are the most common chemotherapies used for AML^12^. Collectively, our data suggest that ALK inhibitor AZD3463 inhibits FLT3-ITD selectively without affecting wild-type FLT3 signaling. While inhibition of FLT3-ITD is beneficial for AML patients, wild-type FLT3 signaling is required for stem and progenitor cell development^4^.

## Supplementary information

Supplementary Materials include "Supplementary materials and methods" and "Supplementary figures (S1 to S7)".
