# Correction: The ALK inhibitor AZD3463 effectively inhibits growth of sorafenib-resistant acute myeloid leukemia

**DOI:** 10.1038/s41408-019-0214-8

**Published:** 2019-07-25

**Authors:** Sausan A. Moharram, Kinjal Shah, Fatima Khanum, Lars Rönnstrand, Julhash U. Kazi

**Affiliations:** 1 Division of Translational Cancer Research, Department of Laboratory Medicine, Lund University, Lund, Sweden; 2 Lund Stem Cell Center, Department of Laboratory Medicine, Lund University, Lund, Sweden; 3 Division of Oncology, Skåne University Hospital, Lund, Sweden

**Keywords:** Acute myeloid leukaemia, Oncogenes

**Correction to: Blood Cancer Journal** 9:5

10.1038/s41408-018-0169-1 published online 15 January 2019

After publication of the original article, the authors realised there was an incorrect image in Fig. S5B. The correct image is reproduced here. The Supplementary File of the original article has also been replaced with this error corrected. This error does not affect the conclusions drawn in the article.

**Fig. S5:**
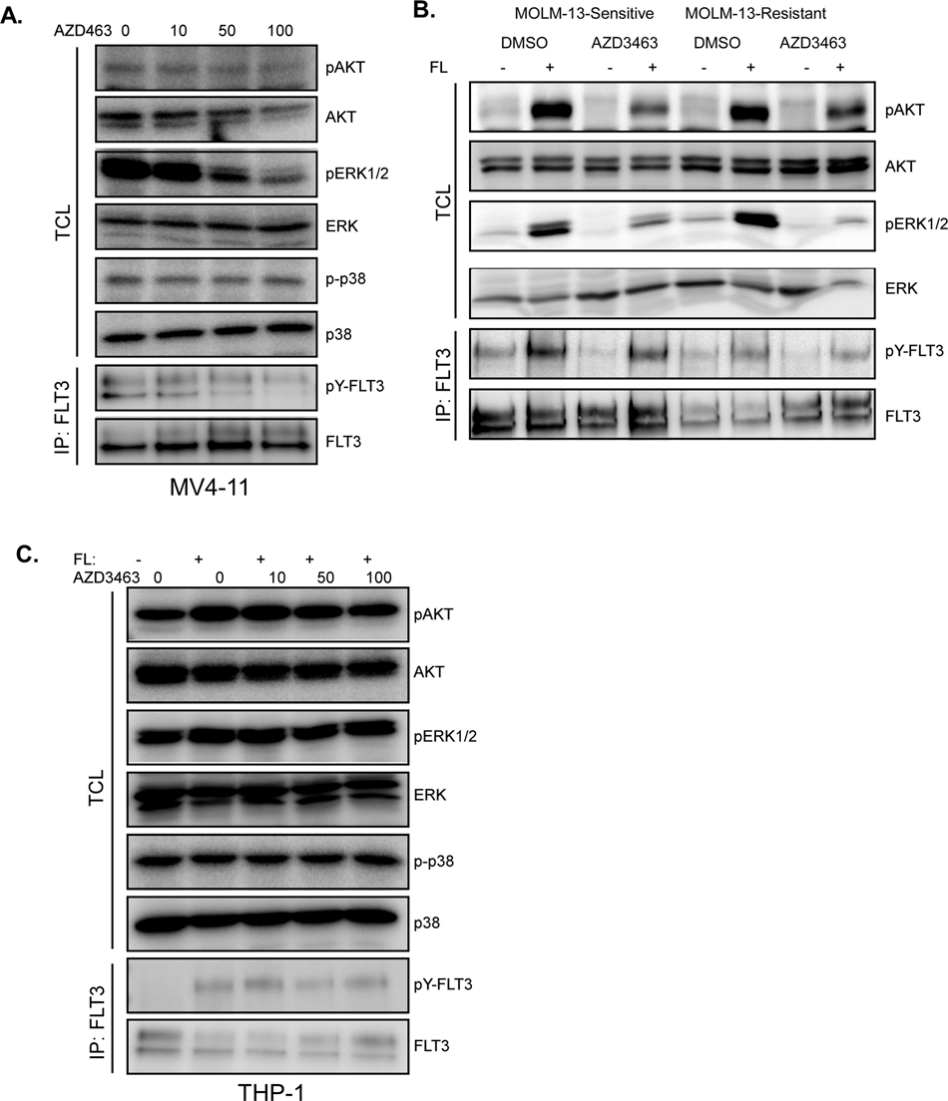
⬛

