# Supplementary material for: The ALK inhibitor AZD3463 effectively inhibits growth of sorafenib-resistant acute myeloid leukemia
Source: Blood Cancer J. 2019 Jan 15;9(2):5. doi: 10.1038/s41408-018-0169-1 (PMC6333797; doi:10.1038/s41408-018-0169-1)
Supplement: Supplementary file 1 — Supplementary Materials include "Supplementary materials and methods" and "Supplementary figures (S1 to S7)". [file 41408_2018_169_MOESM1_ESM.pdf]

The ALK inhibitor AZD3463 effectively inhibits growth of sorafenib-resistant acute myeloid leukemia

Sausan A. Moharram, Kinjal Shah, Fatima Khanum, Lars Rönnstrand and Julhash U. Kazi

Supplementary materials

Supplementary materials and methods:

Reagents and chemicals: The kinase inhibitor library was from Selleck Chemicals (Houston, TX). Cytarabine, Daunorubicin HCl, Vincristine sulfate, Cyclophosphamide, Methotrexate, 6-Mercaptopurine, Doxorubicin HCl and AZD3463 were from MedChemExpress Europe (Sollentuna, Sweden). Dexamethasone was from Sigma Aldrich (St Louis, MI). Sorafenib was from Selleck Chemicals (Houston, TX). Rabbit anti-FLT3 antibody was described earlier [1]. Anti-AKT, anti-ERK, anti-phospho-ERK and anti-beta-actin-HRP were from Santa Cruz Biotechnology (Dallas, TX). Anti-phospho-AKT (S473) was from Abcam. Anti-phospho p38 and anti-p38 were from BD Biosciences. 4G10 was from Millipore. For *in vitro* studies, drugs were dissolved in DMSO and for *in vivo* studies, AZD3463 was first dissolved in DMSO and then diluted using sodium chloride solution before injection.

Cell lines and cell culture and patient materials: The leukemia cell lines Jurkat, MOLM-13, MV4-11, PL21, GDM-1, MOLM-16, NOMO-1, THP-1, KG-1, HL-60 and SKM-1 cells were obtained from Deutsche Sammlung von Mikroorganismen und Zellkulturen (DSMZ, Braunschweig, Germany). All AML cell lines were cultured in RPMI 1640 medium supplemented with 10% heat-inactivated FBS, 100 units/ml penicillin and 100 μg/ml streptomycin. Ba/F3-FLT3-ITD was described previously [2] and Ba/F3-ALK-F1174L was a kind gift from Professor Jianmin Sun. Ba/F3 cells were maintained in RPMI 1640 medium supplemented with 10% heat-inactivated FBS, 10 ng/ml murine IL-3 and 100 units/ml penicillin and 100 μg/ml streptomycin as described elsewhere [3]. FLT3-ITD primary AML cells which are procured from BRCF Biospecimen Repository Core Facility of Kansas University Medical Center, Kansas City, KS and used under ethical permit of BRCF Biospecimen Repository Core Facility of Kansas University Medical Center.

Cell lysis, western blotting, cell viability and apoptosis assays: Cells were lysed in 1% Triton-X 100 lysis buffer supplemented with protease and phosphatase inhibitors. The western blotting procedure was described previously [4]. Cell viability was measured using PrestoBlue (Thermo Fischer) following the protocol provided by the manufacturer. Apoptosis was measured by Annexin V and 7-amino actinomycin D (7-AAD) apoptosis kit (BD Biosciences, Franklin Lakes, NJ).

Peptide-based kinase profiling: PamGene technology for kinase profiling (PamGene, Hertogenbosch, the Netherlands) was used to measure upregulated kinase activities. MOLM-13 cells were serum-starved for 16h before lysis. Tyrosine kinase profiling was performed using standard protocols provided by the manufacturer.

Animal experiment: Ten female NOD scid gamma (NSG) mice each weighing approximately 20 g, (housed by the Laboratory Animal Facilities at Medicon Village, Lund University) were injected subcutaneously with 4 million cells. Mice were then divided into two groups that were either treated with AZD3463 or vehicle. One week after injection of cells, mice were treated six days weekly by intraperitoneal (IP) injection of 15 mg/kg AZD3463 or vehicle for additional seven days. Tumor volume was measured and all mice were sacrificed when one of the tumors reached around 1000 mm^3. The animal experiment was performed under an ethical permit from the Swedish Animal Welfare Authority.

Statistical analysis: All statistical analysis was performed using GraphPad Prism 5.0. For multiple comparisons One-way ANOVA was used. Otherwise, student's t-test was used to compare in between two groups.

Supplementary figures:

Figure S1: Tyrosine kinase profiling in MOLM-13 cells.

Figure S2: Screening the kinase inhibitors library.

Figure S3: Cell viability and apoptosis.

Figure S4: ALK expression in AML cell lines and docking of AZD3463 in the FLT3 kinase domain.

Figure S5: AZD3463 selectively inhibits FLT3-ITD.

Figure S6: AZD3463 and chemotherapy combinations.

Figure S7: AUC for AZD3463 and chemotherapy combinations.

Figure S1: Tyrosine kinase profiling of MOLM-13 cells: Peptide-based kinase profiling shows upregulation of kinase activity of several kinases compared between (A) MOLM-13 sorafenib-sensitive and –resistant, (B) MOLM-13 sorafenib-sensitive and –resistant cells treated with sorafenib, (C) MOLM-13 sorafenib-sensitive cells treated with DMSO and sorafenib and (D) MOLM-13 sorafenib-resistant cells treated with DMSO and sorafenib.

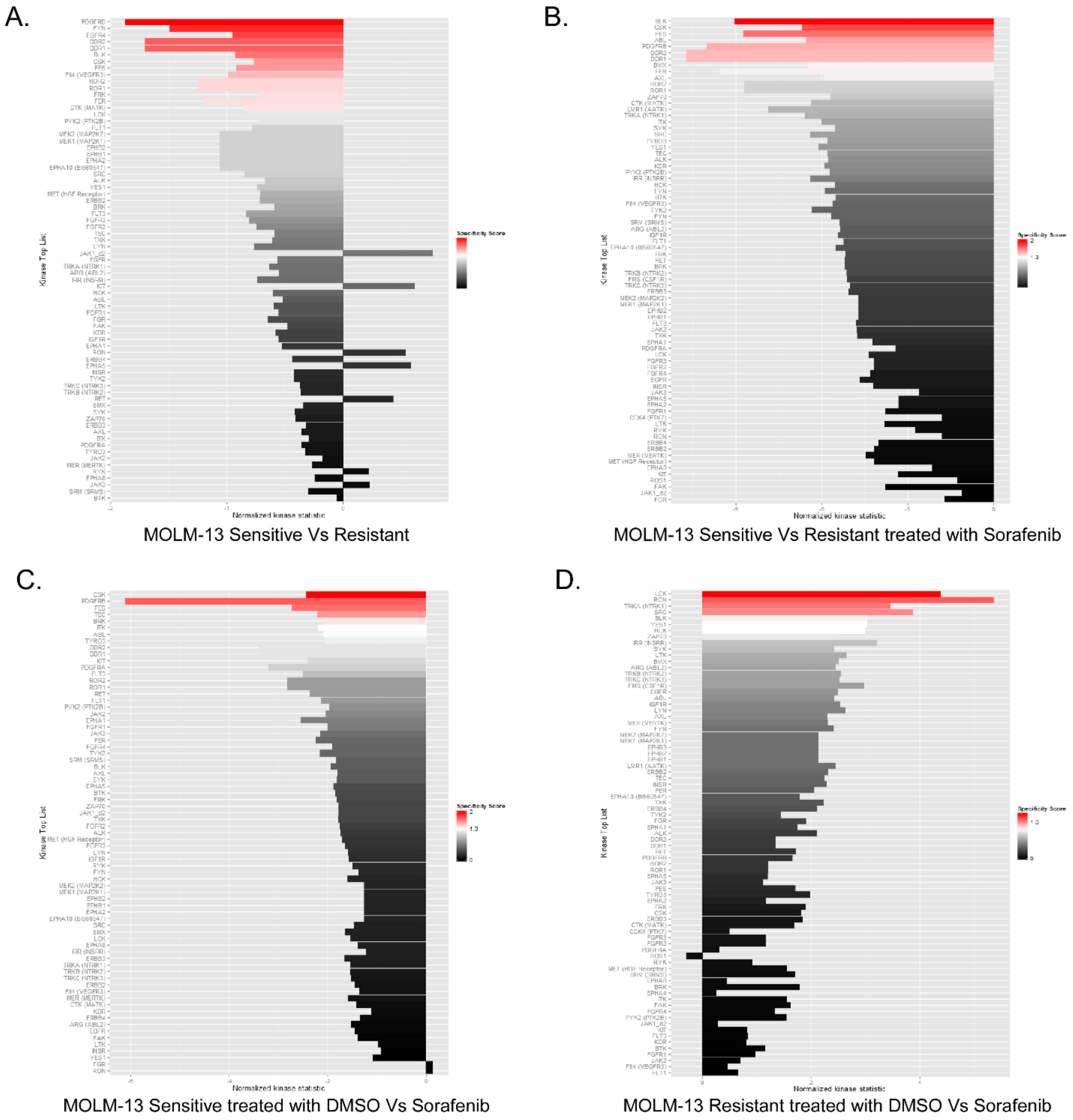

Figure S2: Screening the kinase inhibitor library: MOLM-13 sorafenib-sensitive (A) and –resistant cells (B) were treated with 100 nM concentrations of a panel of kinase inhibitors (378 inhibitors) for two days. Jurkat cells were used as a control. (C-D) Another set of experiment was run using 1000 nM drug concentration. (E) MOLM-13 sorafenib- resistant cells and Jurkat cells were treated with 100 nM of different inhibitors against kinases. Relative cell viability was measured by PrestoBlue after 48h incubation with the drug.

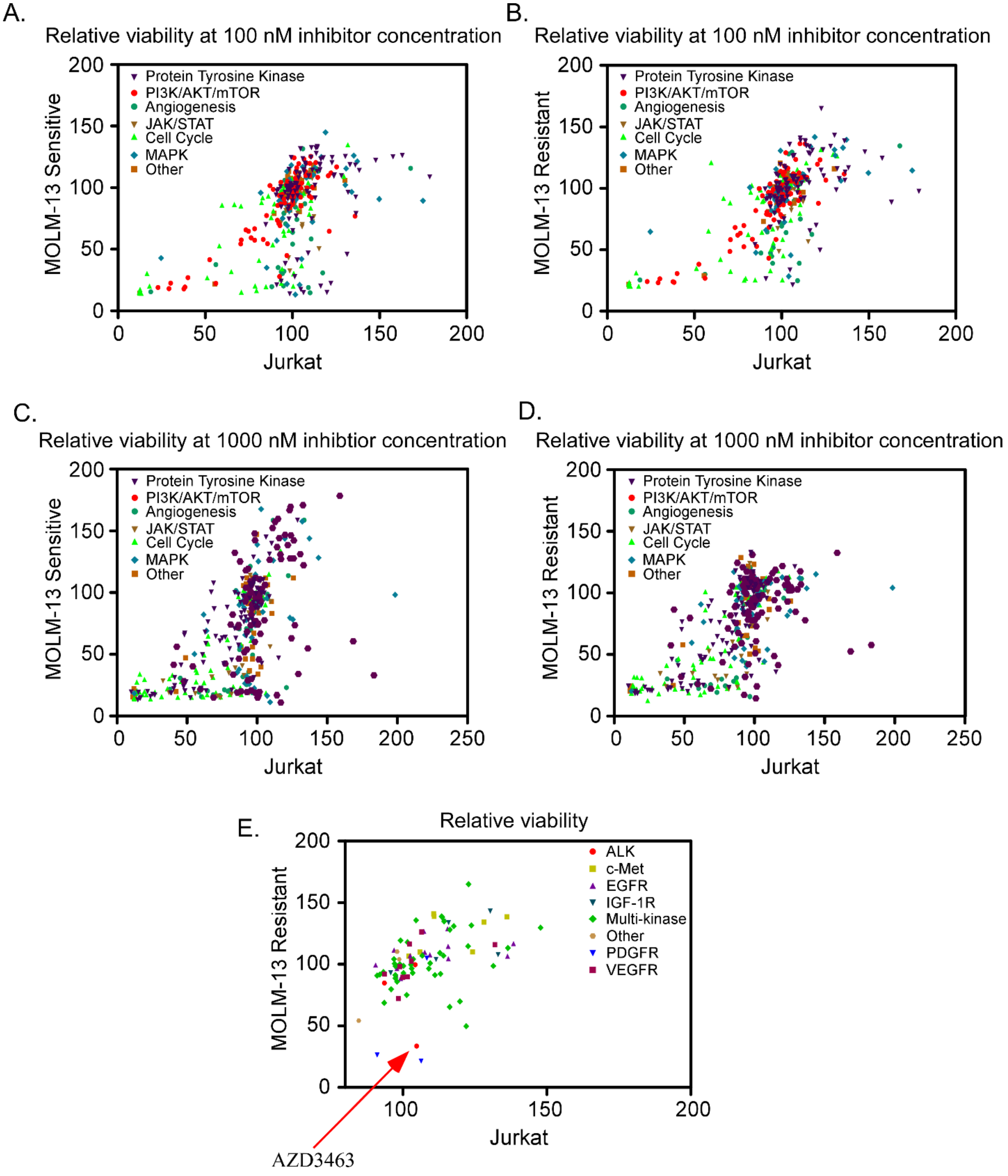

Figure S3: Cell viability and apoptosis: (A) MOLM-13 sorafenib-sensitive and –resistant cells were treated with different concentrations of AZD3463 for two days. Cell viability was measured by PrestoBlue. (B) MOLM-13 sorafenib-sensitive and –resistant cells were treated with 0, 10 nM, 50 nM and 100 nM concentrations of AZD3463 for two days. Cell viability was measured by PrestoBlue. (C) MOLM-13 sorafenib-sensitive and –resistant cells were treated with 0, 1 nM, 10 nM and 100 nM concentrations of AZD3463 for two days. Apoptosis was measured by Annexin V and 7-AAD kit.

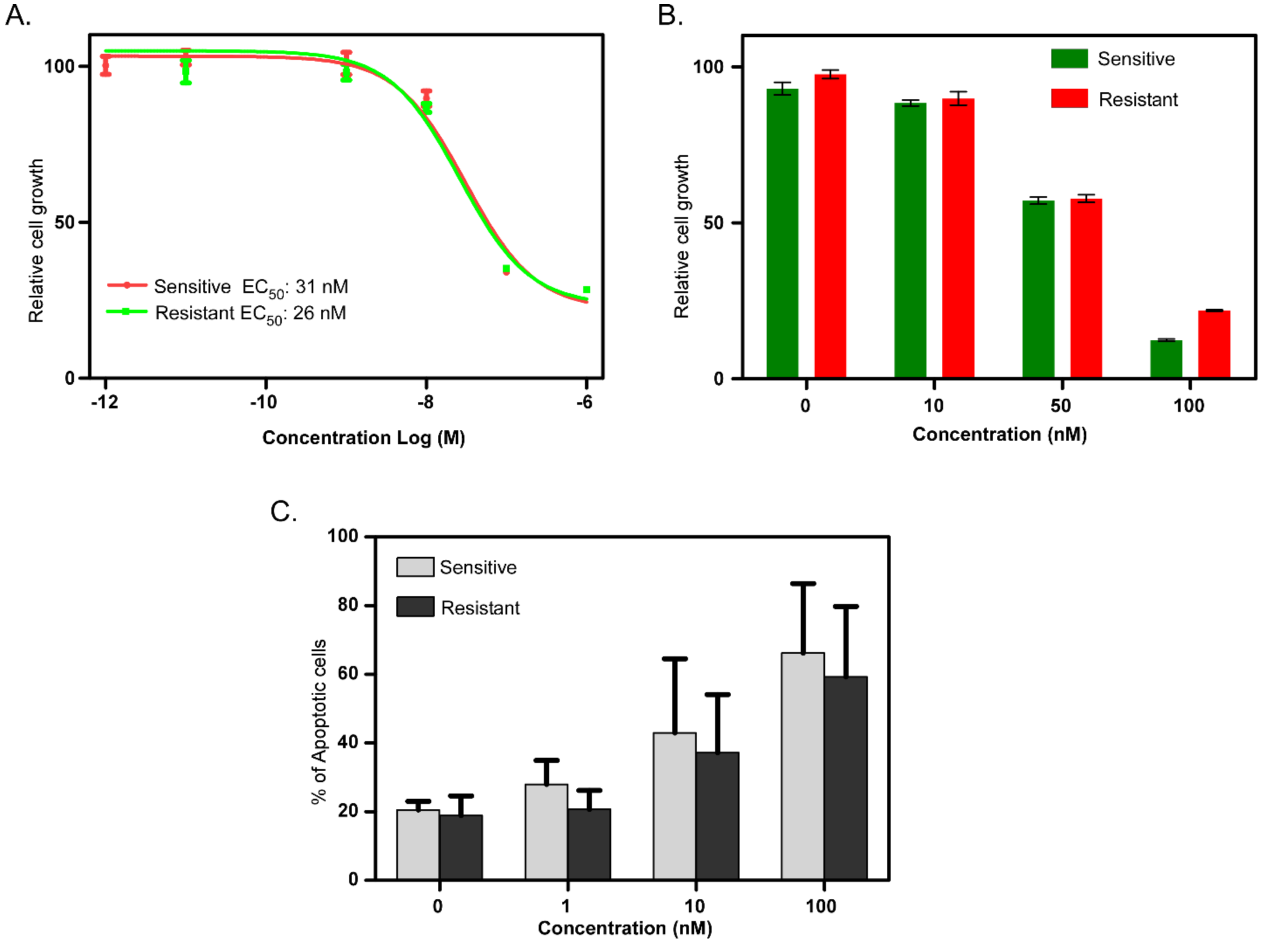

Figure S4: ALK expression in AML cell lines and docking of AZD3463 to the FLT3 kinase domain: (A) Cells were lysed and lysates were used for Western Blotting analysis using anti-ALK and anti-beta-actin antibodies. (B-C) Molecular docking was done by SwissDock online docking server.

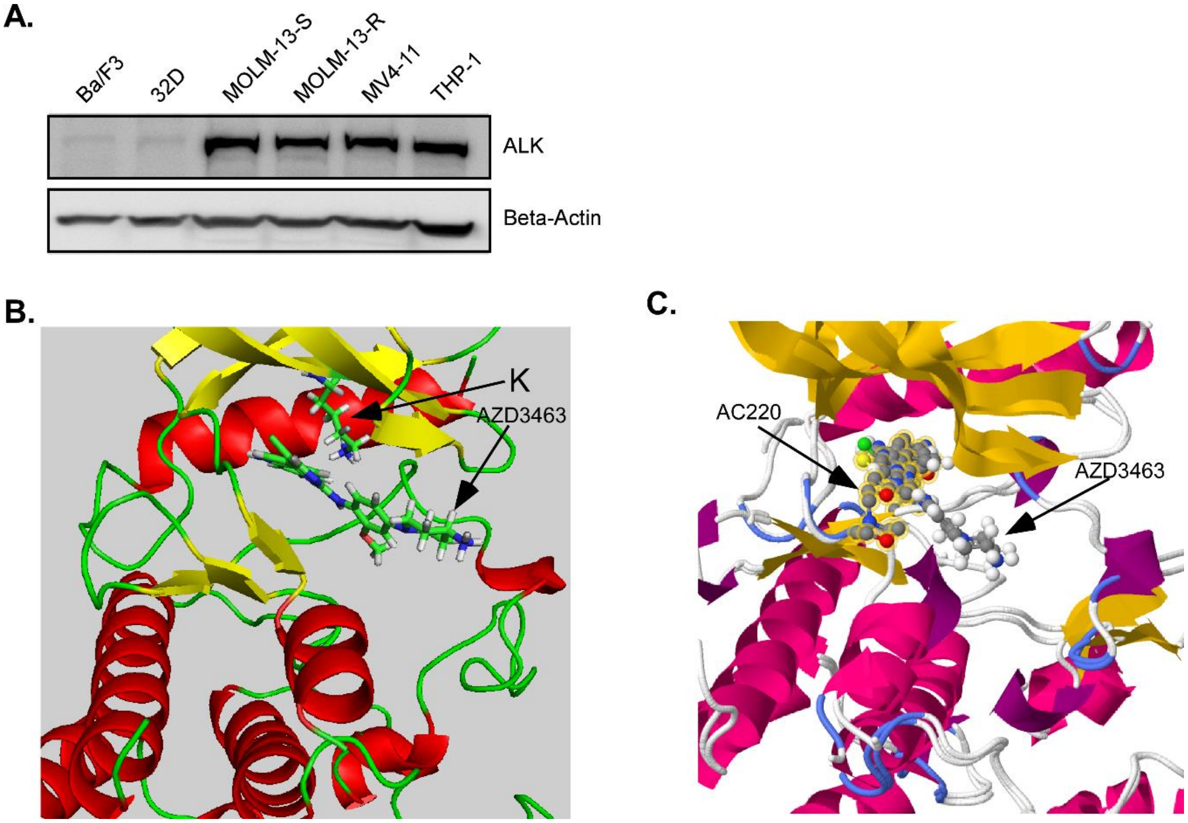

Figure S5: AZD3463 selectively inhibits FLT3-ITD: (A) MV4-11 Cells were treated with increasing concentrations of AZD3463 before lysis. Cell lysates were used to assess phosphorylation of different proteins using phospho-specific antibodies. (B) MOLM-13 sorafenib-sensitive and –resistant cells were treated with AZD3463 for four hours before stimulation with FL. (C) THP-1 cells were treated with AZD3463 for four hours before stimulation with FL.

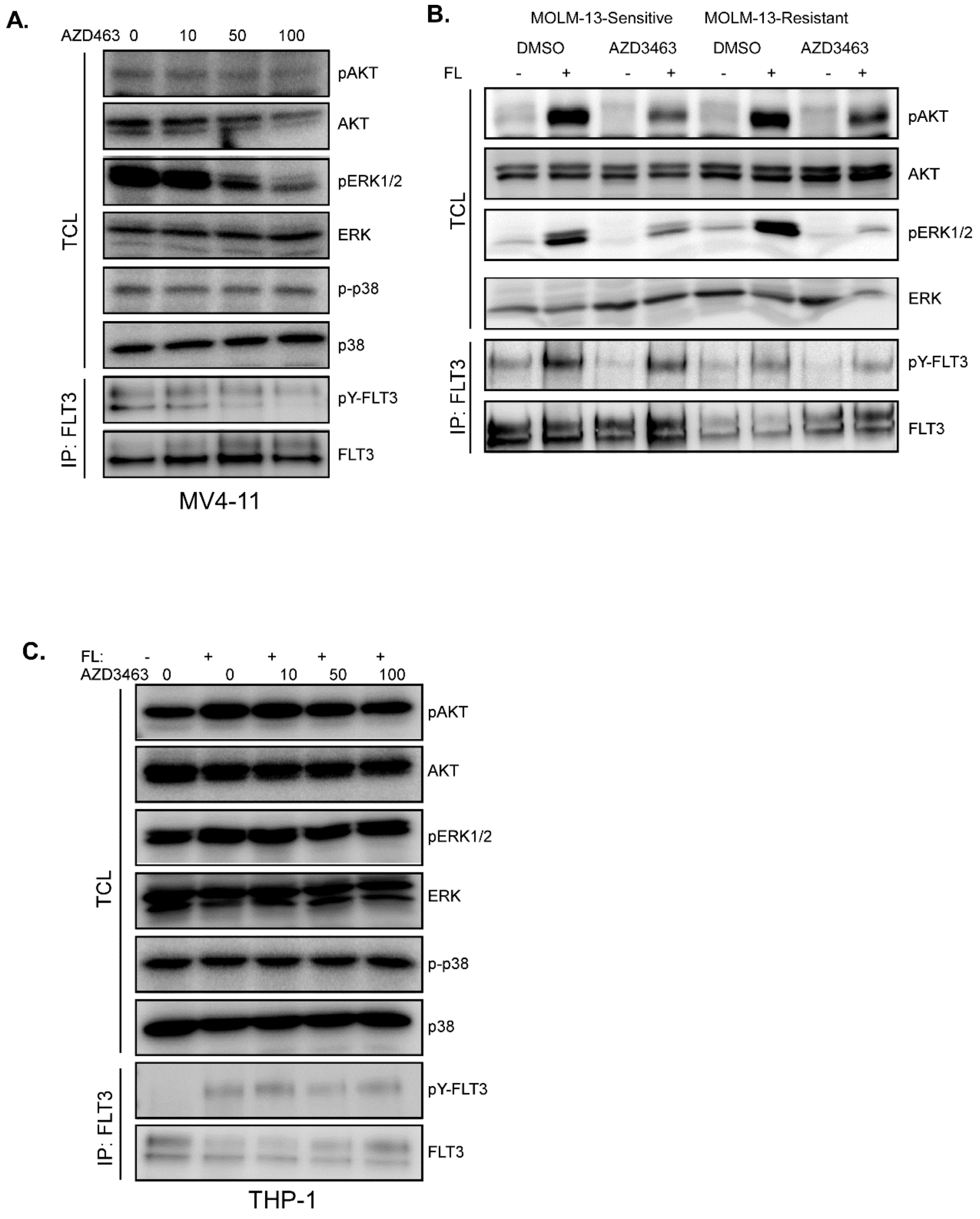

Figure S6: AZD3463 and chemotherapy combinations: MOLM-13, MV4-11 and PL-21 were treated with different concentrations of AZD3436 and various chemotherapeutic agents for two days. PrestoBlue was used to measure cell viability.

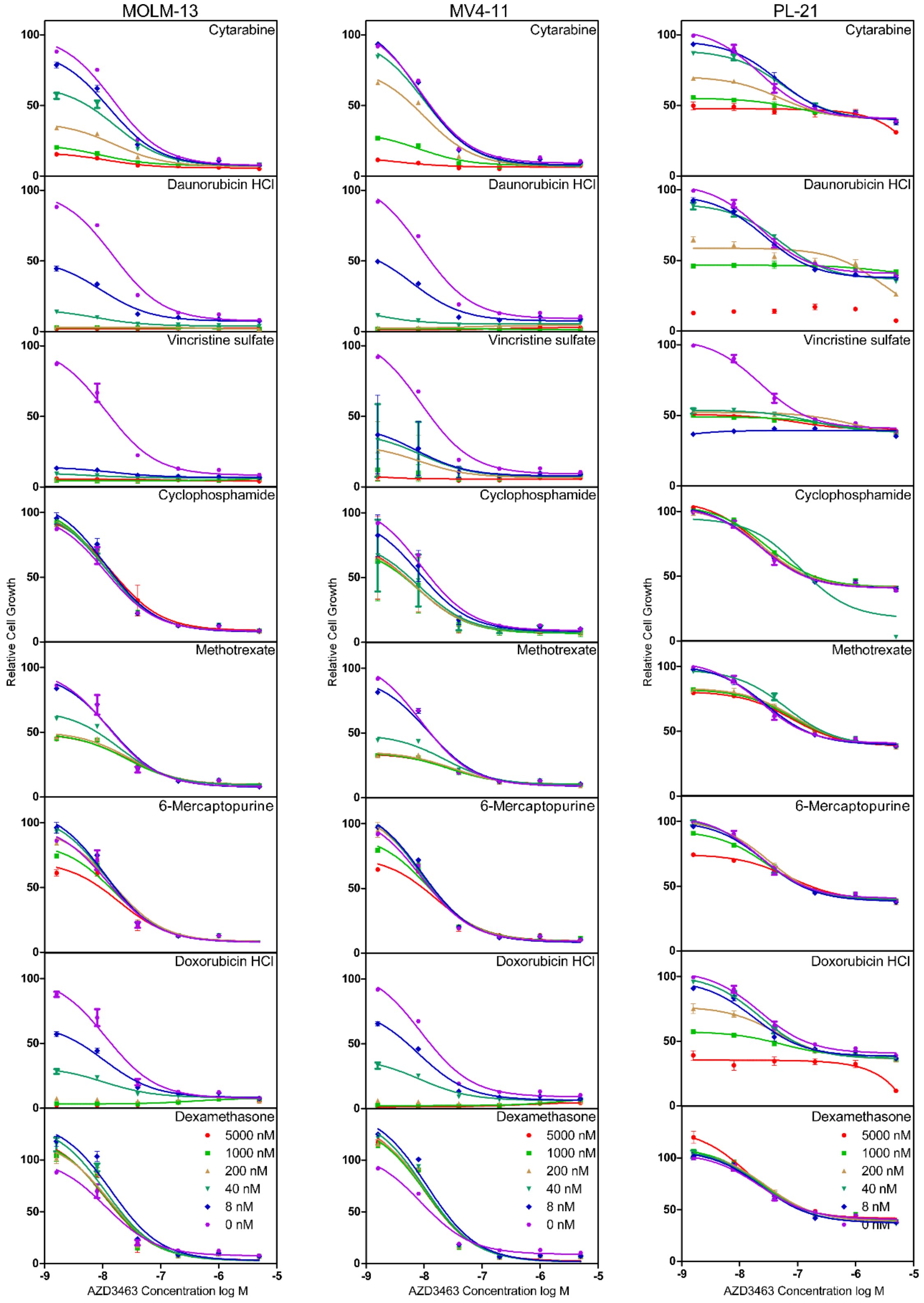

Figure S7: AUC for AZD3463 and chemotherapy combinations: Area under the curve (AUC) was measured from the figure S6 using GraphPad.

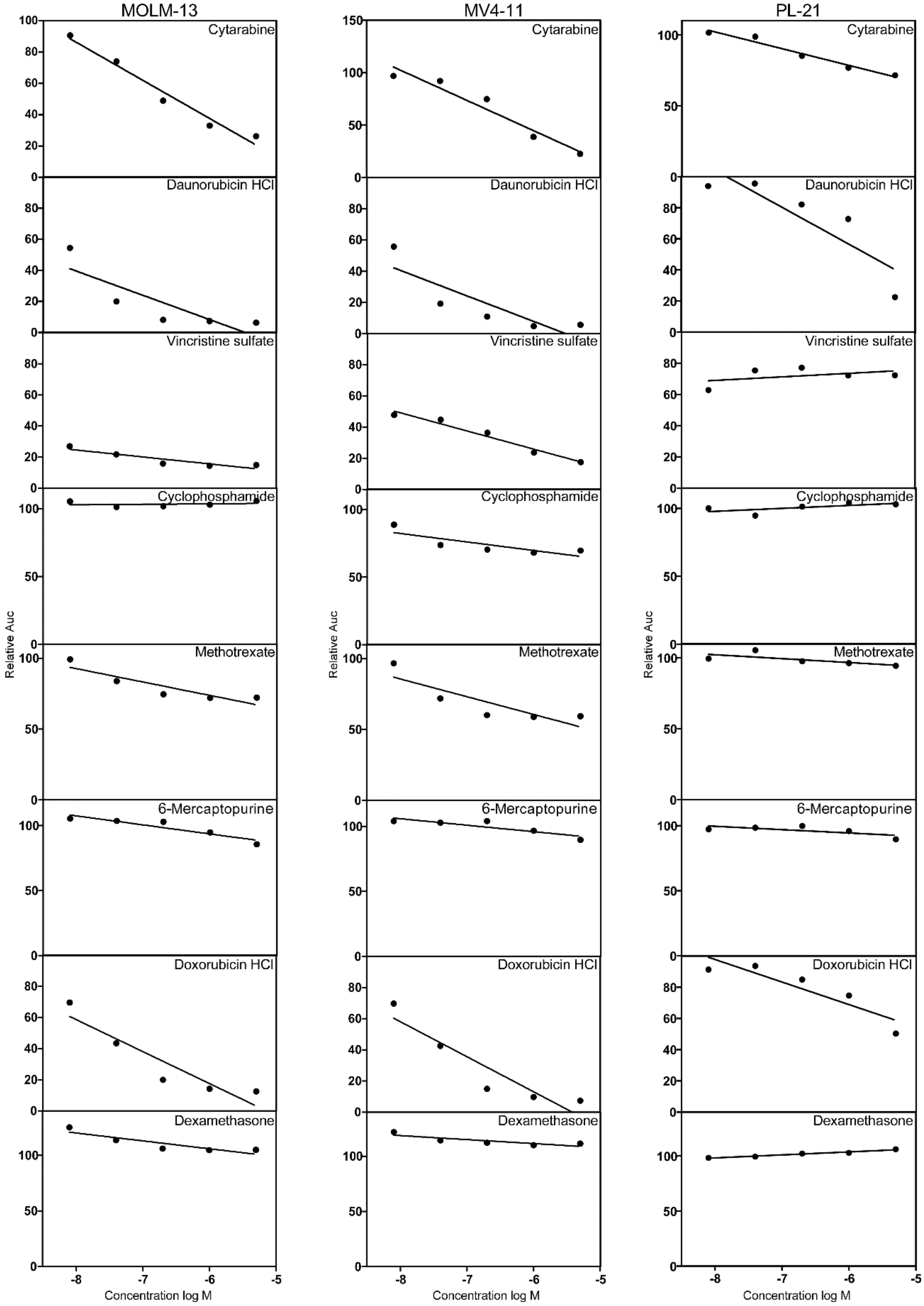

References:

1. Razumovskaya E, Masson K, Khan R, Bengtsson S, Rönnstrand L. Oncogenic Flt3 receptors display different specificity and kinetics of autophosphorylation. *Exp Hematol* 2009 Aug; **37**(8)**:** 979-989.

2. Moharram SA, Chougule RA, Su X, Li T, Sun J, Zhao H, *et al.* Src-like adaptor protein 2 (SLAP2) binds to and inhibits FLT3 signaling. *Oncotarget* 2016 Sep 6; **7**(36)**:** 57770-57782.

3. Kazi JU, Sun J, Rönnstrand L. The presence or absence of IL-3 during long-term culture of Flt3-ITD and c-Kit-D816V expressing Ba/F3 cells influences signaling outcome. *Exp Hematol* 2013 Jul; **41**(7)**:** 585-587.

4. Voytyuk O, Lennartsson J, Mogi A, Caruana G, Courtneidge S, Ashman LK, *et al.* Src family kinases are involved in the differential signaling from two splice forms of c-Kit. *J Biol Chem* 2003 Mar 14; **278**(11)**:** 9159-9166.
